# The Pneumococcal Serine-Rich Repeat Protein Is an Intra-Species Bacterial Adhesin That Promotes Bacterial Aggregation *In Vivo* and in Biofilms

**DOI:** 10.1371/journal.ppat.1001044

**Published:** 2010-08-12

**Authors:** Carlos J. Sanchez, Pooja Shivshankar, Kim Stol, Samuel Trakhtenbroit, Paul M. Sullam, Karin Sauer, Peter W. M. Hermans, Carlos J. Orihuela

**Affiliations:** 1 Department of Microbiology and Immunology, The University of Texas Health Science Center San Antonio, San Antonio, Texas, United States of America; 2 Laboratory of Pediatric Infectious Diseases, Radboud University Nijmegen Medical Centre, Nijmegen, The Netherlands; 3 Division of Infectious Diseases, San Francisco VA Medical Center and the University of California, San Francisco, California, United States of America; 4 Department of Biological Sciences, Binghamton University, Binghamton, New York, United States of America; University of Pennsylvania, United States of America

## Abstract

The Pneumococcal serine-rich repeat protein (PsrP) is a pathogenicity island encoded adhesin that has been positively correlated with the ability of *Streptococcus pneumoniae* to cause invasive disease. Previous studies have shown that PsrP mediates bacterial attachment to Keratin 10 (K10) on the surface of lung cells through amino acids 273–341 located in the Basic Region (BR) domain. In this study we determined that the BR domain of PsrP also mediates an intra-species interaction that promotes the formation of large bacterial aggregates in the nasopharynx and lungs of infected mice as well as in continuous flow-through models of mature biofilms. Using numerous methods, including complementation of mutants with BR domain deficient constructs, fluorescent microscopy with Cy3-labeled recombinant (r)BR, Far Western blotting of bacterial lysates, co-immunoprecipitation with rBR, and growth of biofilms in the presence of antibodies and competitive peptides, we determined that the BR domain, in particular amino acids 122–166 of PsrP, promoted bacterial aggregation and that antibodies against the BR domain were neutralizing. Using similar methodologies, we also determined that SraP and GspB, the Serine-rich repeat proteins (SRRPs) of *Staphylococcus aureus* and *Streptococcus gordonii*, respectively, also promoted bacterial aggregation and that their Non-repeat domains bound to their respective SRRPs. This is the first report to show the presence of biofilm-like structures in the lungs of animals infected with *S. pneumoniae* and show that SRRPs have dual roles as host and bacterial adhesins. These studies suggest that recombinant Non-repeat domains of SRRPs (i.e. BR for *S. pneumoniae*) may be useful as vaccine antigens to protect against Gram-positive bacteria that cause infection.

## Author Summary

Serine-rich repeat proteins (SRRPs) are a family of surface-expressed proteins found in numerous Gram-positive pathogens, including *Staphylococcus aureus, Streptococcus pneumoniae*, Group B streptococci, and the oral streptococci that cause infective endocarditis. For all of these bacteria, SRRPs have been demonstrated to play pivotal roles in adhesion to tissues and the development of invasive disease. It is now known that biofilm formation is an important step for bacterial pathogenesis. Bacteria in biofilms have been shown to have differences in metabolism, gene expression, and protein production that contribute to enhanced surface adhesion and the persistence of an infection. Herein we describe a novel role for PsrP, the *S. pneumoniae* SRRP, as an intra-species bacterial adhesin that promotes bacterial aggregation in the lungs of infected mice during pneumonia. *In vitro* we show that the Basic Region domain of PsrP promotes self-interactions that result in denser biofilms, greater biofilm biomass, and altered architectures of surface grown cultures; these interactions could be neutralized by antibodies to PsrP that are protective against pneumococcal infection. We also demonstrate that the SRRPs of *S. aureus* and *Streptococcus gordonii* also function as intra-species bacterial adhesins. Therefore we conclude that SRRPs have dual roles as host-cell and intra-species bacterial adhesins.

## Introduction

*Streptococcus pneumoniae* is a leading cause of otitis media (OM), community-acquired pneumonia, sepsis and meningitis. Primarily a commensal, *S. pneumoniae* typically colonizes the nasopharynx asymptomatically, however in susceptible individuals such as infants, the elderly, persons who are immunocompromised, and those with sickle cell anemia, the pneumococcus is often able to cause opportunistic diseases [1], [2], [3], [4]. Worldwide, *S. pneumoniae* is responsible for up to 14.5 million episodes of invasive pneumococcal disease (IPD) and 11% of all deaths in children [5], [6]. In the elderly the mortality-rate associated with IPD can exceed 20% and for those in nursing homes may be as high as 40% [7]. Thus, the pneumococcus has been and remains a major cause of morbidity and mortality.

*psrP-secY2A2* is a *S. pneumoniae* pathogenicity island whose presence has been positively correlated with the ability to cause human disease [8]. Analyses of the published *S. pneumoniae* genomes has demonstrated that *psrP-secY2A2* is present and conserved in a number of globally distributed invasive clones, in particular those belonging to serotypes not covered by the heptavalent conjugate vaccine [9]. To date, numerous studies have shown that deletion of genes within *psrP-secY2A2* attenuated the ability of *S. pneumoniae* to cause disease in mice. *psrP-secY2A2* mutants were shown to be unable to attach to lung cells, establish lower respiratory tract infection, and were delayed in their ability to enter the bloodstream from the lungs. Importantly, the same studies found that *psrP-secY2A2* did not play an important role during nasopharyngeal colonization or during sepsis following intraperitoneal challenge [10], [11], [12], [13]. Thus *psrP-secY2A2* is currently understood to be a lung-specific virulence determinant.

**Figure 1:**
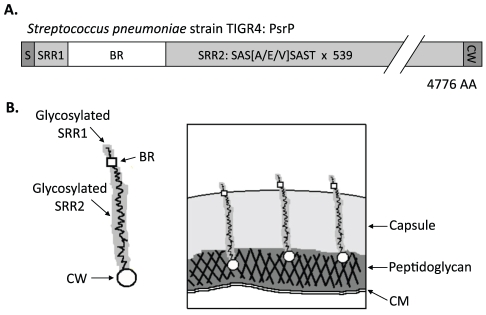
Hypothetical model of PsrP on the surface of *S. pneumoniae*. **A**) Domain structure of PsrP: N-terminal signal peptide (S); serine-rich repeat motif 1 (SAS[A/E/V]SAST X 11) (SRR1); basic region (BR); serine-rich repeat motif 2 (SRR2); and the cell wall anchoring domain (CW) at the C-terminus. **B**) Illustration of PsrP on the bacterial surface. Based on the structural organization of PsrP and studies demonstrating that the BR domain binds to K10 on lung cells [13], we propose that the CWAD attaches the protein to the cell wall, while the long glycosylated SRR2 domain serves to extend BR through the capsular polysaccharide to mediate interactions.

In TIGR4, a virulent serotype 4 laboratory strain, *psrP-secY2A2* is 37-kb in length and encodes 18 proteins. These include the Pneumococcal serine-rich repeat protein (PsrP), which is a lung cell adhesin, 10 putative glycosyltranferases, and 7 proteins homologous to components of an accessory Sec translocase [14]. To date, the latter 17 genes remain uncharacterized; however, based on their homology to genes found within the Serine-rich repeat protein (SRRP) locus of *Streptococcus gordonii*, the encoded proteins are putatively responsible for the intracellular glycosylation of PsrP and for its transport to the bacterial surface [8], [15], [16], [17], [18]. PsrP in TIGR4 is composed of 4,776 amino acids, has been confirmed to be glycosylated, and separates at an apparent molecular mass of 2,300 kDa on an agarose gel [13]. It is one of the largest bacterial proteins known. PsrP is organized into multiple domains including a cleavable N-terminal signal peptide, a small serine-rich repeat region (SRR1), a unique non-repeat region (NR), followed by a second extremely long serine-rich region (SRR2), and a C-terminal cell wall anchor domain containing an LPXTG motif (Figure 1A). The SRR1 and SRR2 domains of PsrP are composed of 8 and 539 serine-rich repeats (SRR) of the amino acid sequence SAS[A/E/V]SAS[T/I], respectively, and are the domains believed to be glycosylated. The NR domain of PsrP has a predicted pI value of 9.9, for this reason it is called the Basic Region (BR) domain.

*S. pneumoniae* is surrounded by a polysaccharide capsule that protects the bacteria from phagocytosis but also inhibits adhesion to epithelial cells [19]. Based on the size and domain organization of PsrP we have previously hypothesized that the extremely long SRR2 domain serves to extend the BR domain through the capsular polysaccharide to mediate lung cell adhesion (Figure 1B) [12], [13]. Consistent with this model, we have previously shown that PsrP is expressed on the bacterial surface, that the BR domain, in particular amino acids 273–341, was responsible for PsrP-mediated adhesion to Keratin 10 (K10) on lung cells, and that complementation of *psrP* deficient mutants with a truncated version of the protein (having only 33 SRRs in its SRR2 domain) restored the ability of uncapsulated but not capsulated PsrP mutants to adhere to A549 cells, a human type II pneumocyte cell line [13].

It is now recognized that biofilms play an important role during infectious diseases. Briefly, bacteria in biofilms are more resistant to host-defense mechanisms including phagocytosis and serve as a recalcitrant source of bacteria during antimicrobial therapy [20], [21]. For *S. pneumoniae*, pneumococcal biofilms have been shown to occur in the middle ears of children with chronic otitis media and is thought to contribute to its refractory nature [22]. Likewise, biofilms have been detected in the nasopharynx of infected chinchillas [23]. However, until now biofilm structures have not been described in the lungs during pneumococcal pneumonia. This is in contrast to other respiratory tract pathogens, such as *Pseudomonas aeruginosa* and *Bordatella pertussis*, for which *in vivo* biofilm production is now recognized to be an important pathogenic mechanism [21]. Herein, we demonstrate for the first time that *S. pneumoniae* forms biofilm-like aggregates in the lungs. We show that this phenomenon is PsrP-dependent and mediated by its BR domain. Using recombinant protein and SRRP mutants, we show that the SRRPs of *S. gordonii* and *Staphylococcus aureus*, GspB and SraP, respectively, also promote bacterial aggregation, thus describing a previously unrecognized role for members of the SRRP family. Collectively, these findings suggest an important dual role for PsrP and other SRRPs during infection, host cell and intra-species bacterial adhesion, both of which may be targeted for intervention with antibodies against recombinant (r)NR.

## Results

### PsrP promotes pneumococcal aggregation *in vivo*

**Figure 2:**
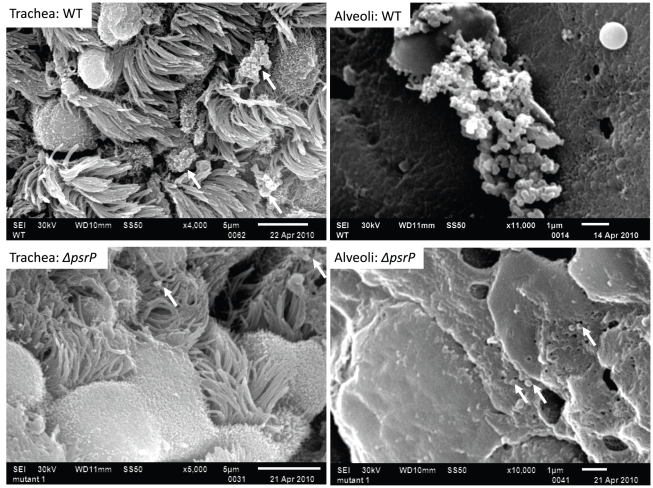
PsrP promotes the formation of bacterial aggregates *in vivo*. SEM images of bronchial and alveolar epithelial cells following infection with TIGR4 (WT) and T4 *ΔpsrP (ΔpsrP)*. White arrows point at attached bacteria. Note the presence of WT bacteria in large aggregates of various sizes.

**Figure 3:**
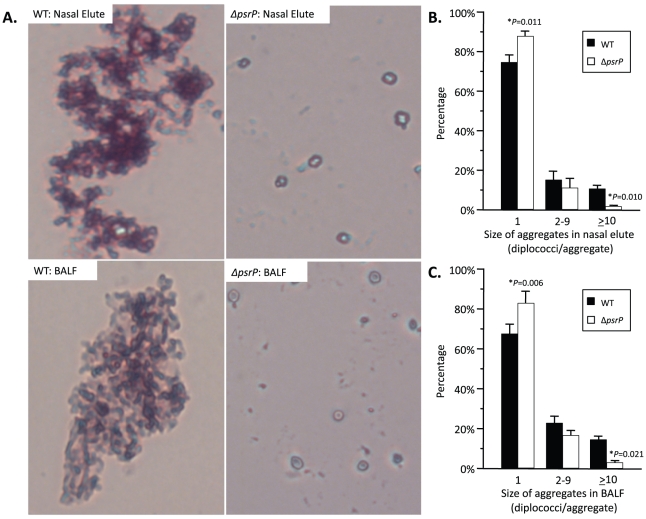
Frequency of bacterial aggregates in the nasopharynx and lungs. **A**) Micrographs of TIGR4 (WT) and T4 *ΔpsrP (ΔpsrP)* Gram-stained bacteria from either BAL or nasal lavage (IN) elutes. Images were taken at 400× magnification. Images are not representative of the total bacteria population, but instead are shown to demonstrate the typical pneumococcal aggregate containing at least 10 or more individual diplococci (10+). **B**) Actual percentages of pneumococcal aggregate based on size in the nasopharynx and **C**) lungs following counting of >100 randomly selected CFUs per biological replicate. Note that TIGR4 had significantly greater levels of 2–9 and 10+ aggregates compared to T4 *ΔpsrP*. Furthermore, while 10+ aggregates were observed in mice infected with T4 *ΔpsrP*, albeit infrequently, the largest of these aggregates were not comparable in size to those formed by TIGR4. Statistical analyses were performed using a Student's *t*-test.

To test whether PsrP contributed to biofilm or microcolony formation *in vivo* mice were infected with TIGR4 and its isogenic *psrP* deficient mutant, T4 *ΔpsrP*, and whole lung sections were examined using scanning electron microscopy (SEM). As would be expected for both wild type and the mutant, the majority of bacteria present were in the form of diplococci. However, for TIGR4 we also observed the presence of large bacterial aggregates attached to ciliated bronchial epithelial cells as well as to alveolar epithelial cells (Figure 2). For quantitative analysis of this phenomenon, nasal lavage fluid and bronchoalveolar lavage (BAL) fluid from mice was collected two days post-challenge. Aliquots from each biological sample were heat-fixed to glass slides, Gram-stained, and examined with a microscope (Figure 3A). In all, the number of bacterial aggregates composed of 2–9, and ≥10 diplococci were significantly greater for mice infected with TIGR4 than T4 *ΔpsrP* in both the nasopharyngeal and BAL elute fluids (Figure 3B,C). Moreover, the largest aggregates, those composed of >100 bacteria, were observed only in mice infected with TIGR4. Fluorescent imaging of bacteria in frozen lung sections confirmed this phenotype; large bacterial aggregates were only detected in the lungs of TIGR4 infected mice (Figure S1). Thus we determined that PsrP promoted the formation of biofilm-like aggregates *in vivo*, including in the nasopharynx, a site previously shown not to require PsrP for bacterial colonization [12].

### PsrP affects intimate bacteria to bacteria interactions

**Figure 4:**
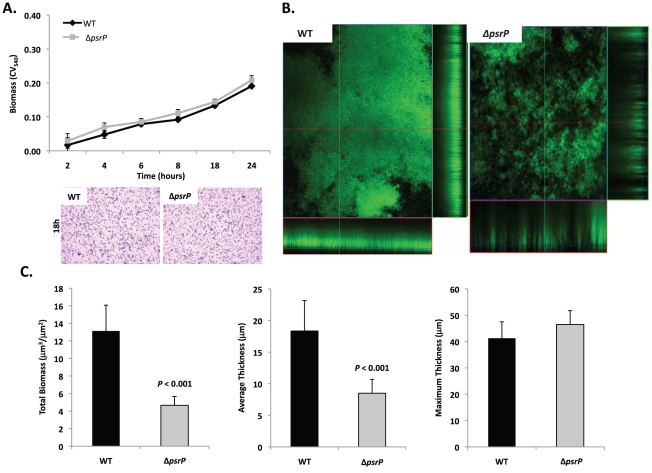
Deletion of *psrP* alters bacterial interactions in mature biofilms but not during early biofilm attachment. **A**) Attachment of TIGR4 (WT) and T4 *ΔpsrP (ΔpsrP)* to the bottom of 96-well polystyrene microtiter plate in an early biofilm model. Biofilm biomass was determined using crystal violet (CV_540_) stain as described in the methods. **B**) Micrographs of mature TIGR4 and T4 *ΔpsrP* biofilms grown in a flow cell under once-through flow conditions for 3 days. Bacteria were visualized with Live/Dead BacLight stain using an inverted confocal laser scanning microscope at 400× magnification. **C**) Quantitative analysis of the biofilms was performed using COMSTAT image analysis software. All experiments were performed in triplicate. Statistical analyses were performed using a two-tailed Student's *t*-test. For panel C error bars denote standard error.

Given the previous results, moreover to develop an *in vitro* model that was amendable to manipulation, the ability of TIGR4 and T4 *ΔpsrP* to form early biofilms was tested using microtiter plates [24]. As shown in Figure 4A, no differences were observed between wild type and the mutant, suggesting that PsrP does not play a role in pneumococcal attachment to polystyrene or the formation of early biofilm structures, in particular the bacteria lawn. The role of PsrP was next tested in 3-day old mature biofilms using the once-through continuous flow cells as described previously by Allegrucci *et al.* [25]. In this system, a stark difference in the architecture of TIGR4 and T4 *ΔpsrP* biofilms was observed (Figure 4B). Wild type biofilms displayed a dense cloud-like morphology with extremely large aggregates that covered the glass surface. Closer inspection revealed that these aggregates were composed of tightly clustered pneumococci. In contrast, T4 *ΔpsrP* biofilms displayed a less intimate phenotype characterized by smaller aggregates, gaps, and the formation of columns, resulting in an overall patchier phenotype. Quantitative analysis of the biofilm structures using COMSTAT software confirmed that TIGR4 biofilms had significantly greater total biomass and average thickness than those formed by the T4 *ΔpsrP* (Figure 4C). No differences in either the maximum thickness of the biofilms or the roughness coefficient (a measure of biofilm heterogeneity) were observed (Figure 4C; data not shown, respectively), indicating that T4 *ΔpsrP* could still form biofilms, although with distinct architecture. Importantly, T4 *ΩpsrP-secY2A2*, a mutant deficient in the entire *psrP-secY2A2* pathogenicity island, behaved identically to T4 *ΔpsrP*, forming patchy biofilms with small aggregates and less intimate associated bacteria (Figure S2).

**Figure 5:**
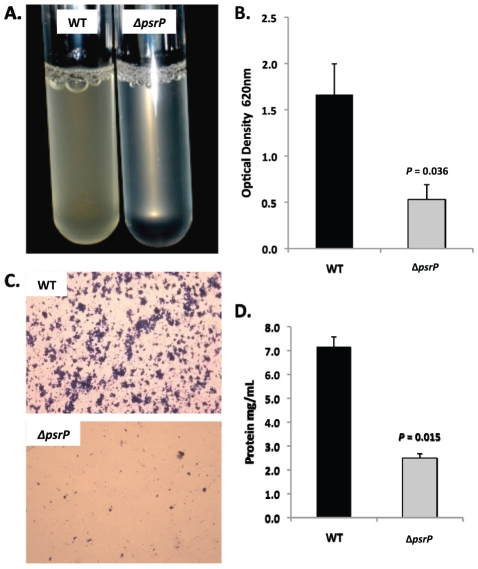
PsrP promotes bacterial aggregation in a line biofilm model. Mid-logarithmic growth phase TIGR4 (WT) and T4 *ΔpsrP (ΔpsrP)* were used to inoculate 1 meter of a 0.8 mm diameter silicone-lined plastic tubing. After 3 days, biofilms within the lines were extruded. **A**) Representative photograph of the exudate suspension immediately following its collection. **B**) Optical density (OD_540_) of bacterial exudates. **C**). Microscopic images of CV stained bacteria extracted from the lines. Note the formation of aggregates by TIGR4 but not T4 *ΔpsrP*. **D**) Levels of protein in bacteria line exudates as determined by bicinchoninic acid assay (BCA) following detergent lysis of the bacteria. Images are representative of at least 3 experiments. Statistical analyses were performed using a two-tailed Student's *t*-test. Error bars denote standard error.

Bacterial biofilms were also grown under once through conditions in silicone tubing. After a designated time, the biofilms were extruded from the line and examined for biomass both visually and quantitatively. After 3 days of growth, differences between TIGR4 and T4 *ΔpsrP* in opacity of the exudates were visible to the eye (Figure 5A) and could be confirmed using a spectrophotometer which showed a >3-fold difference in optical density (Figure 5B). Microscopic visualization of the line exudates following crystal violet (CV) staining revealed that TIGR4 had formed large aggregates whereas T4 *ΔpsrP* exudates were composed of small clusters or of individual diplococci (Figure 5C). Increased biofilm biomass was supported by measurement of total protein concentrations that showed TIGR4 biofilm exudates had 2–3 fold more protein than those corresponding to T4 *ΔpsrP* (Figure 5D).

Of note, during planktonic growth TIGR4, T4 *ΔpsrP*, and T4 *ΩpsrP-secy2A2* were indistinguishable, growing either as short chains or diplococci with a marked absence of aggregates (data not shown). This led us to examine *psrP* transcription using Real-Time PCR and the finding that TIGR4 expressed *psrP* at levels 47-fold greater during biofilm versus planktonic culture (*P* = 0.04 using a Student's *t*-test). Thus low expression of *psrP* may be one reason TIGR4 did not form aggregates during liquid culture.

### The BR domain mediates intra-species bacterial interactions

To date a number of groups, including our own, have shown that SRRPs mediate bacterial adhesion to host cells primarily through their NR domain [13], [26], [27]. For this reason we sought to test whether the BR domain of PsrP was also involved in biofilm/bacterial aggregation. To do this we first utilized a pre-existing collection (described in Figure S3) of encapsulated (T4 *ΩpsrP*) and unencapsulated (T4R *ΩpsrP*) *S. pneumoniae* mutants deficient in PsrP that either expressed a truncated version of PsrP with 33 SRRs in its SRR2 domain (PsrP_SRR2(33)_), a similar truncated version lacking the BR domain (PsrP_SRR2(33)-BR_), or carried the empty expression vector pNE1 [13]. These strains were tested for their ability to form biofilms in silicone lines under once through conditions.

**Figure 6:**
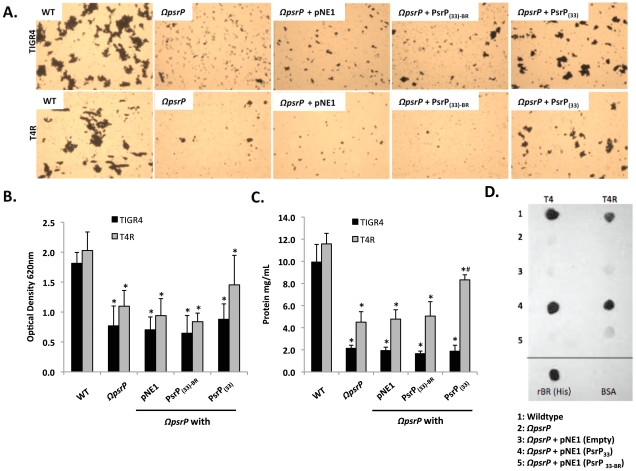
The BR domain of PsrP mediates intra-species bacterial interactions. Encapsulated and unencapsulated mutants of TIGR4, lacking PsrP (T4 *ΩpsrP*, T4R *ΩpsrP*, respectively), were complemented with plasmids expressing: a truncated version of PsrP having only 33 SRR2 repeats (PsrP_(33)_), the truncated version of PsrP missing the BR domain (PsrP_(33)-BR_), or with the empty expression vector (pNE1). **A**) Microscopic images of CV stained bacteria isolated from the line biofilm model. **B**) Optical density (OD_620_) of biofilm line exudates. **C**) Biomass of the biofilms as determined by protein levels using the BCA assay. **D**) Far Western analyses of recombinant BR interactions with membrane-bound truncated versions of PsrP expressed in *S. pneumoniae*. All images are representative of at least 3 independent experiments. Statistical analyses were performed using 1-Way ANOVA analysis. Error bars denote standard error. For panel B and C asterisks denote statistical significance versus WT; hash sign denotes statistical significance versus the empty vector control.

Complementation of T4 *ΩpsrP* with PsrP_SRR2(33)_, but not PsrP_SRR2(33)-BR_ or the empty pNE1 vector, partially restored the ability of T4 *ΩpsrP* to form large aggregates in the lines when examined microscopically (Figure 6A). However, measurement of other biofilm markers such as optical density and total protein concentration showed no differences between any of the complemented mutants and the negative controls (Figure 6B–C). Complementation of T4R *ΩpsrP* with PsrP_SRR2(33)_, also partially restored the ability of T4R *ΩpsrP* to form aggregates (Figure 6A). In this instance, line exudates from T4R *ΩpsrP* with PsrP_SRR2(33)_ had significant more biofilm biomass than the negative controls (Figure 6B–C). Importantly, the truncated version of PsrP lacking the BR domain failed to restore, even partially, T4 *ΩpsrP* or T4R *ΩpsrP* suggesting that the BR domain was responsible for the intra-species aggregation. This was subsequently confirmed by Far-Western blot analyses that showed that Gst-tagged recombinant BR (Gst-BR) bound only to *S. pneumoniae* cell lysates that contained a truncated PsrP with the BR domain (Figure 6D) and a control experiment showing that a Gst-tagged *Chlamydia trachomatis* protein did not interact with these lysates (Figure S4).

**Figure 7:**
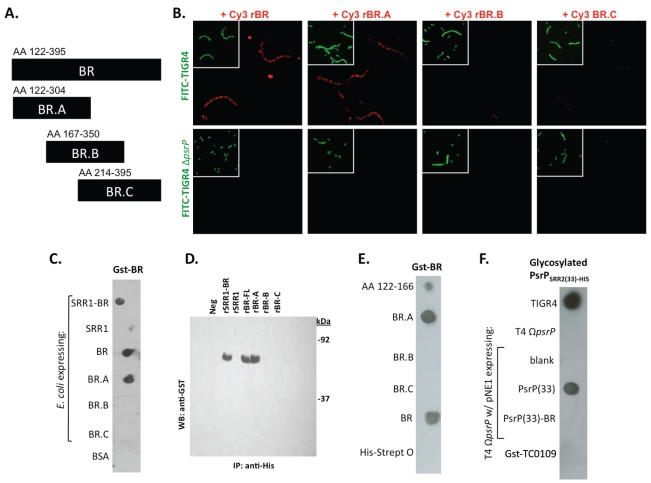
Recombinant BR interacts with pneumococci that carry amino acids 122–166 of PsrP. **A**) The designated recombinant PsrP constructs were expressed and purified from *E. coli*. **B**) Micrographs of FITC-labeled bacteria following their incubation with CY3-labeled rBR or the designated truncated versions. Note that only Cy3-labeled rBR and rBR.A bound to TIGR4. Moreover, neither bound to T4 *ΔpsrP*. This suggests that recombinant BR binds to PsrP on the bacteria surface. **C**) Far Western blot examining the interaction of Gst-BR with cell lysates from *E. coli* expressing assorted rBR constructs spotted on a membrane. **D**) Co-immunoprecipitation of Gst-BR (65 kDa) from spiked *E. coli* lysates expressing His-tagged rBR constructs. **E**) Far Western blot examining the interaction of Gst-BR with purified rBR constructs and a synthesized peptide corresponding to AA 122–166. **F**) Far Western blot examining the interaction of a glycosylated PsrP construct purified from *S. pneumoniae*, PsrP_SRR2(33)-HIS_ with glycosylated PsrP constructs expressed in TIGR4.

To further explore the role of the BR domain in the observed bacteria to bacteria interactions, the ability of His-tagged BR constructs (rBR; Figure 7A), purified from *Escherichia coli* and Cy3 labeled, were tested for their ability to bind to the surface of TIGR4 and T4 *ΔpsrP*. Full-length rBR interacted with TIGR4 but not with T4 *ΔpsrP* (Figure 7B), confirming not only that PsrP bound to pneumococci, but also suggesting that its ligand was another PsrP. Furthermore, only rBR.A retained the ability to attach to PsrP on the pneumococcal surface. This suggested that the binding domain of PsrP was possibly located within AA 122–166, the section not shared between rBR.A and rBR.B.

Hereafter, BR to BR interactions were tested for by Far Western and co-immunoprecipitation. Far Western blot experiments using assorted *E. coli* cell lysates from bacteria expressing assorted rBR constructs, confirmed that only lysates containing PsrP constructs with AA 122–166 bound successfully to Gst-BR (Figure 7C). This was also observed in co-immunoprecipitation experiments, whereby Gst-BR was tested for its ability to bind whole cell lysates from *E. coli* expressing versions of PsrP (Figure 7D). Far Western blots using purified proteins showed that Gst-BR had affinity to purified rBR, rBR.A, and a synthesized peptide corresponding to AA 122–166, but not rBR.B, BR.C, or the control his-tagged Streptolysin O (Figure 7E). Hence, using numerous assays it was determined that the BR domain, most likely AA 122–166, had self-interacting properties that might be responsible for the observed bacterial aggregation.

Of note, because the BR constructs were purified from *E. coli* and PsrP is normally glycosylated, the above observations may have been an artifact of the unglycosylated constructs used. To address this possibility a glycosyated truncated PsrP construct was purified from *S. pneumoniae* (PsrP_SRR2(33)-HIS_; Figure S5) and tested for its ability to bind *S. pneumoniae* cell lysates containing either native PsrP or assorted constructs. As shown in Figure 7F, it was determined that a glycosylated PsrP probe maintained specificity for the BR domain even in the context of glycosylated recipient protein. A finding that supports the notion that PsrP to PsrP interactions occur in natural setting when PsrP is always glycosylated.

### The aggregation and K10-binding subdomains of BR are independent

**Figure 8:**
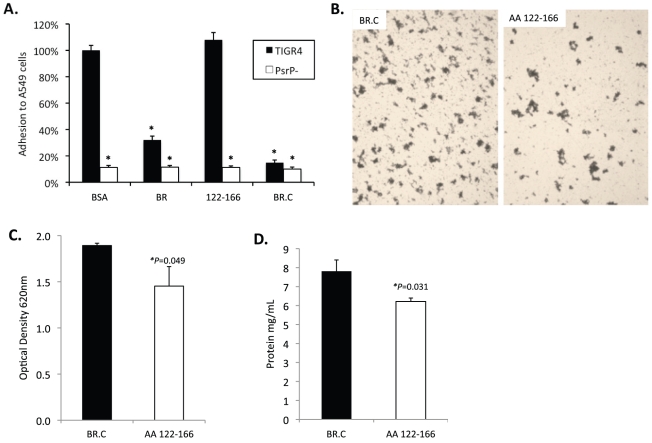
Incubation of bacteria with AA 122–166 impairs bacterial aggregation but not adhesion to cells. **A**) Adhesion of TIGR4 following pre-incubation of A549 cells with media containing 1.0 µM of the designated rBR constructs. All values were normalized against cells incubated with BSA (163±4 bacteria/10^6^ cells). TIGR4 in media containing 1 µM BR.C or the synthesized peptide 122–126 was used to inoculate and grow biofilms in the line biofilm model. After 3 days, biofilms within the lines were extruded. **B**) Representative photograph of the exudate suspension immediately following its collection and staining with CV. Images are representative of at least 3 experiments. **C**) Optical density (OD_540_) of bacterial exudates. **D**) Levels of protein in bacteria line exudates. Statistical analyses were performed using a Student's *t*-test. Error bars denote standard error.

To determine whether the BR aggregation (AA 122–167) and the K10 binding subdomains (AA 273–341) of BR had functionally independent roles, competitive inhibition assays were performed using rBR constructs. Bacterial adhesion to A549 cells was tested following incubation of cells with the AA 122–166 peptide, rBR, and rBR.C (Figure 8A). Pre-treatment of A549 cells with AA 122–167 had no impact on adhesion. In contrast and consistent with the location of the K10 binding domain within BR.C: 1) TIGR4 adhered significantly less to cells treated with rBR or rBR.C, 2) TIGR4 adhered to BSA treated cells better than T4 Δ*psrP*. In complementary biofilm experiments the opposite result was observed. Addition of 1 µM peptide AA 122–167 to media reduced the aggregation phenotype observed for TIGR4 (Figure 8B) and modestly lowered the optical density of the biofilm exudate and the total biomass collected from the continuous flow lines versus addition of BR.C (Figure 8C–D). Thus these findings suggested that the aggregation and K10 subdomains of PsrP had distinct roles that did not overlap during host cell adhesion or biofilm formation.

Finally we sought to determine a biological effect for the aggregation phenotype. We observed that after 1 hour, 69±2% of J477 macrophages incubated with planktonically grown TIGR4 were associated with FITC-labeled bacteria whereas only 51±5% of macrophages mixed with biofilm grown TIGR4 were positive (*P* = 0.024). Macrophages exposed to biofilm grown TIGR4 also took up less bacteria than macrophages mixed with planktonic (74±1%; *P* = <0.001) and biofilm (60±1%; *P* = <0.001) cultures of T4 Δ*psrP*. Interestingly, a 10% reduction in macrophage uptake was observed for the biofilm versus planktonic grown T4 Δ*psrP* cultures (*P* = 0.077); and no difference was observed between macrophage uptake of TIGR4 and T4 Δ*psrP* when taken from planktonic cultures. These findings suggest, that in addition to PsrP, other bacterial factors expressed during growth in a biofilm also affect opsonophagoyctosis.

### Antibodies to the BR domain, but not to the SASASAST motif, block bacterial aggregation

**Figure 9:**
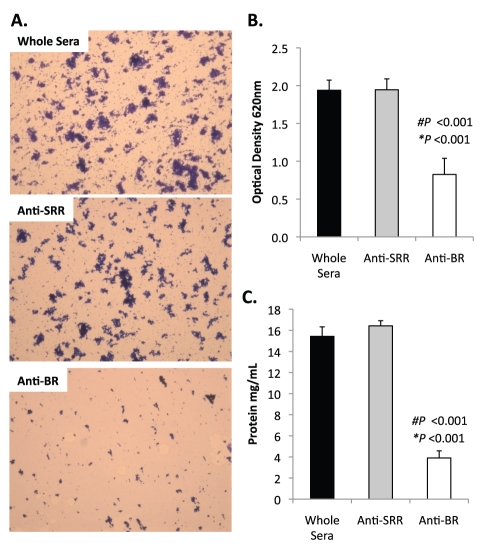
Antibodies to the BR domain but not the serine-rich motif block intra-species bacterial interactions. THB was supplemented with either a 1∶1000 dilution of naïve rabbit serum (control), rabbit antiserum from rabbits immunized with a SASASASTSASASAST peptide designed after the SRR motif, or rabbit antiserum to recombinant BR. **A**) Micrographs of CV stained bacteria extruded from the biofilm lines. **B**) Optical density of bacterial exudates. **C**) Levels of protein in bacterial line exudates as determined by BCA analysis. Images are representative of at least 3 independent experiments. Statistical analyses were performed using a two-tailed Student's *t*-test. Number sign denotes statistical significance versus whole serum. Asterisks denote statistical significance versus anti-SRR serum. Error bars denote standard error.

Previously we had shown that antibodies against the SRR1-BR domains of PsrP neutralized the ability of *S. pneumoniae* to attach to lung cells and that vaccination with rBR protected mice against pneumococcal challenge [12], [13]. For this reason we tested the ability of polyclonal antiserum against rBR and against a SRR motif peptide to block bacterial aggregation in the biofilm line model. Todd Hewitt Broth (THB) supplemented with a 1∶1000 dilution of antiserum against the BR domain inhibited the formation of bacterial aggregates as observed by microscopic visualization of the biofilm line exudates. In contrast, bacteria in media supplemented with antiserum to the SRR motif peptide or that from naïve animals, formed aggregates similar to wild type bacteria grown under serum free conditions (Figure 9A). Biofilm exudate optical density and protein concentrations supported these microscopic observations (Figure 9B–C). To determine whether the effect of the BR antiserum on biofilm formation was specific for TIGR4, we tested the ability of antibodies to the BR domain to block biofilm formation in unrelated clinical isolates (Figure S6). Antiserum against rBR from TIGR4 inhibited biofilm formation in two unrelated clinical isolates that carried PsrP. The same sera had no effect on biofilm formation by an invasive serotype 14 isolate that lacked PsrP. Therefore these studies confirmed previous observations that increased bacteria aggregation in biofilm models can occur independently of PsrP, but that if present, antiserum against BR can block the contribution of PsrP to these processes.

### SRRPs mediate intra-species adhesion in pathogenic bacteria

**Figure 10:**
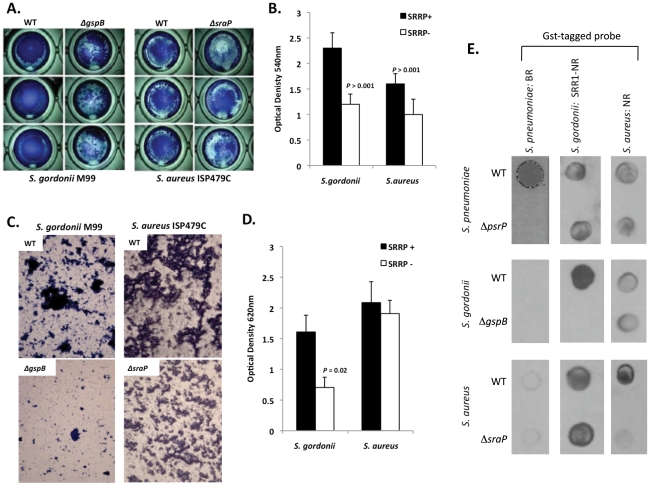
The SRRPs of *S. gordonii* and *S. aureus* promote bacterial aggregation. Light microscopic images of CV stained **A**) *S. gordonii M99* and **B**) *S. aureus ISP479C* and their respective isogenic SRRP mutants following 24 hours of growth in a 96-well polystyrene microtiter plate early biofilm model. **B**) Average biomass of early biofilms as determined by CV_540_ analyses. Error bars denote standard deviation. **C**) Microscopic images of bacteria extruded from the biofilm lines after 3 days for *S. gordonii* and 1 day for *S. aureus*. **D**) Measurement of optical density of bacterial exudates collected from the biofilm lines. Error bars denote standard deviation. **E**) Far-western examining the intra- and inter-species specificity of the NR domains of *S. pneumoniae*, *S. gordonii* and *S. aureus*. Whole cell lysates from *S. gordonii*, *S. aureus*, and their respective isogenic SRRP mutants were spotted onto nitrocellulose membranes and probed with these GST-tagged proteins. Images are representative of three individual experiments. For panels **B**) and **D**) statistical analyses were performed using a Student's *t*-test.

To determine whether other SRRPs also mediated intra-species aggregation we tested the effect of *gspB* and *sraP* deletion on *S. gordonii* and *S. aureus* biofilm architecture, respectively. Deletion of *gspB* and *sraP* negatively impacted biofilm formation in the microtiter biofilm model at 24 hours (Figure 10A,B). Growth of wild type and mutant bacteria in the line models also demonstrated that both proteins contributed to the formation of large aggregates during surface attached growth; although this property was much more dramatic for *S. gordonii* than for *S. aureus* which did not show a significant difference in the optical densities of the exudates (Figure 10C,D). Of note, *S. aureus* biofilm experiments were stopped after 1 day due to bacteria overgrowth and blockage of the lines.

Subsequent Far Western analysis using Gst-BR from *S. pneumoniae* as well as recombinant SRR1-NR from SraP and recombinant NR from GspB showed that these proteins have affinity for cell lysates from their parent strain but not for cell lysates from isogenic SRRP deficient mutants (Figure 10E). This supports the notion that these proteins might be involved in intra-species aggregation. For PsrP BR from *S. pneumoniae*, no affinity was observed for cell lysates from either *S. gordonii* or *S. aureus* suggesting that PsrP does not play a role as an inter-species adhesin (Figure 10E). In contrast, the NR constructs from *S. aureus* and *S. gordonii* bound to cell lysates from the other bacteria, even in the absence of the SRRP (Figure 10E). The discrepancy between PsrP and the other SRRPs might be explained by the fact that certain SRRPs have been described to have lectin activity [26], [27]. In contrast PsrP adhesion has been shown to be independent of lectin-activity [13].

## Discussion

To date, SRRPs have been described in at least 9 Gram-positive bacteria and have been shown to function as adhesins that contribute to virulence. For example, deletion of *sraP* and *gspB* in *S. aureus* and *S. gordonii*, respectively, decreased the ability of these bacteria to bind to platelets and form vegetative plaques on heart valves of catheterized rats [27], [28]. Similarly, Srr-1 of *Streptococcus agalactiae* has been shown to bind human Keratin 4, mediate adherence to mucosal epithelial cells, and promote invasion of bacteria through human brain microvasculature endothelial cells [29], [30]. SRRPs also mediate acellular attachment, a role important for colonization of the dental surface by oral streptococci. Froelinger and Fives-Taylor showed that *Streptococcus parasanguis* containing mutations of Fap1 failed to attach to saliva-coated hydroxyapatite [31]. Likewise, deletion of *srpA* significantly diminished the ability of *Streptococcus cristatus* to attach to glass slides [32]. Thus, while it was well established that SRRPs play an important role in bacterial attachment to cells or surfaces, until this report their role as intra-species adhesins remained unrecognized.

A dual role, host and bacterial adhesin for bacterial surface proteins is not unprecedented. For example, in *Streptococcus pyogenes* and *S. agalactiae*, the pilus proteins mediate adhesion to epithelial cells and promote microtiter biofilm formation [33], [34]. Likewise, for *Neisseria meningitidis*, PilX, also a pilus-associated protein, mediates adhesion to epithelial cells and facilitates bacterial aggregation [35]. For the pneumococcus, some evidence existed that bacterial adhesins may also have dual roles. In 2008, Munoz-Elias *et al.* found that the pneumococcal adhesins Choline binding protein A and the pilus protein RrgA were both required for robust biofilm formation on microtiter plates and efficient nasopharyngeal colonization [36]. However, the attenuated biofilm phenotype was observed only with unencapsulated bacteria and encapsulated mutants formed biofilms normally. Other pneumococcal proteins shown to affect biofilm formation *in vitro* include Neuraminidase A, which possibly alters the extracellular matrix [37], [38], [39], competence proteins, which suggest an altered protein profile [40], [41], and capsule synthesis enzymes, which were determined to be down regulated in biofilms [36], [42], [43]. Unlike PsrP, which would be expected to bridge cells directly, these proteins most likely act indirectly by altering gene expression, the extracellular milieu, or the surface availability of other adhesins, including possibly RrgA and CbpA.

Our studies determined that the self-aggregating subdomain of PsrP was located in the BR domain and involves amino acids 122–166. Recombinant BR constructs containing these amino acids were able to bind *S. pneumoniae* carrying PsrP, had an affinity for the BR domain in other PsrP constructs, and could modestly inhibit biofilm formation when added to media. Importantly, adhesion assays using pretreated cells and biofilm assays with rBR.C showed that the AA 122–166 was not responsible for adhesion to lung cells and that the K10 binding subdomain (AA 273–341) was not involved in bacterial aggregation. Thus these subdomains appeared to have independent roles during the conditions tested. Further studies are warranted to delineate the specific AAs responsible for these adhesive properties, also to determine the structure of the BR domain and clarify how these subdomains interact with PsrP on other pneumococci and K10 on lung cells.

GspB and SraP have been previously shown to bind platelets [27], [28]. While the ligand for SraP is unknown, it has been determined that GspB binds to Sialyl T-antigen on platelet membrane glycoprotein Ibα [26], [27]. The observation that the NR domains of GspB and SraP bound to cell lysates containing their respective SRRPs but not to their mutants and that the mutants had diminished aggregative properties suggests that SRRPs in other bacteria might also mediate aggregation *in vivo*. One could imagine that SraP on *S. aureus* or GspB on *S. gordonii* mediating attachment to platelets and cells in an endocarditic lesion while at the same time mediating adhesion of individual bacteria to each other. Similarly, one could envision a microcolony of the pneumococcus in the lungs with some bacteria attached to host cells via PsrP/K10 interactions and other bacteria attached to these bacteria through PsrP/PsrP interactions. Presumably, this is what was observed in the lungs of the infected mice. Interestingly, the finding that GspB and SraP NRs bound to cell lysates from other bacteria suggests that these proteins may also mediate inter-species biofilm formation. For *S. gordonii*, this would be relevant as the dental plaque is now recognized to be a multi-species biofilm. Importantly, neutralization of pneumococcal aggregation in biofilms with BR antiserum suggests that SRRPs might have utility as vaccine antigens. One caveat is that SRRPs would have to be one-component of a multi-valent vaccine because not all strains of *S. pneumoniae*, *S. aureus*, or the oral streptococci carry these proteins.

In previous studies we had found that the length of the SRR2 domain was important for adhesion to K10 when capsule was present. Consistent with these findings, the inability of truncated PsrP to fully complement capsulated mutants supports our hypothetical model that the SRR2 domain serves to extend the BR domain away from the cell to mediate bacterial interactions. This model is also indirectly supported by Munoz-Elias *et al.*, who showed that down-regulation of capsule allowed CbpA and RrgA to contribute to biofilm production [36]. It is also noteworthy to state that Munoz-Elias *et al.* did not identify PsrP in their screen for biofilm mutants although they used TIGR4 which carries PsrP. This can be explained by the fact that we observed no contribution for PsrP in the microtiter plate early biofilm model.

We observed that PsrP-mediated bacterial aggregation occurred in the nasopharynx, despite earlier studies demonstrating that K10 was absent from this site and that PsrP was not required for nasopharyngeal colonization. Aggregation of *S. pneumoniae* in the nasopharynx may serve as a mechanism to resist opsonophagocytosis as shown herein, or we speculate a way to resist desiccation during transmission of infectious particles. The observation that aggregates were present at an anatomical site that lacked K10, further supports an independent role for these PsrP subdomains. In regards to opsonophagoyctosis, one important consideration is that the pneumococcus most likely has different gene expression profiles in vivo as an aggregate attached to a cell versus in vitro as a biofilm [44]. Thus caution is warranted in applying our vitro observations, such as resistance to opsonophagoyctosis or enhanced PsrP expression during biofilm growth, with events that occur in vivo.

Polyclonal antibodies against the BR domain, but not the SRR motif, neutralized the ability of TIGR4 and clinical isolates carrying PsrP to form aggregates in the line model. These findings were consistent with previous studies showing that antibodies against BR also neutralized its ability to mediate adhesion to host cells and protected mice against pneumonia [12], [13]. One possible reason that antibodies against the SRR motif peptide failed to have a neutralizing effect is that PsrP is glycosylated and antibodies against the peptide failed to recognize the native version of the protein. Alternatively, antibodies to the SRR motif may bind away from the BR domain and therefore do not inhibit the ability of the BR domain to self-interact. Interestingly, polyclonal antibodies to surface proteins often promote aggregation. This did not occur for unknown reasons. Finally, our finding that antibodies against rBR neutralized bacterial aggregation in the biofilm line model suggests that the same antibodies might also neutralize bacterial aggregation *in vivo*. This remains to be tested, however, the protection that was observed in mice following immunization with rBR [13], may have been in part due to inhibition of bacterial aggregation in addition to blocking interactions with K10.

Importantly, because rNR domains produced in *E. coli* are not glycosylated, yet for the tested SRRPs were able to aggregate, immunoprecipitate, and bind to native protein in cell lysates, it seems that the BR domain does not require glycosylation to function as a self-adhesin. This is supported by the observation that addition of antibodies against unglycosylated rBR and that synthetic peptide AA 122–166 both inhibited bacterial aggregation in the biofilm line. In contrast to the latter concept, Wu *et al.* demonstrated that monoclonal antibodies specific for the glycan motifs of the serine-rich repeat motifs of Fap1 were capable of blocking attachment to saliva coated hydroxyapatite by *Streptococcus parasanguis* [45]. Importantly, Fap1 is the most divergent of the SRRPs and has 2 NR domains. Fap1 adhesion to saliva coated hydroxyapatite is mediated by glyconjugates on the serine-rich repeat domain [46]; as evidenced by the fact that inactivation of one of the glycosyltranferases known to modify the glycan moieties of Fap1, drastically altered the ability of *S. parasanguis* to form biofilms [45]. Thus Fap1 is interesting because it suggests an NR-independent mechanism for SRRP adhesion, which is distinct from those discussed for GspB, SraP, or PsrP. Future studies need to further examine the differences between these diverse SRRPs and to determine if the two NRs of Fap1 play a role in bacterial aggregation. This is especially true given that the NR domain of SraP has a pI of 5.6, in contrast to the basic NRs of GspB (9.5 pI) and PsrP (9.9 pI) [47].

In summary, we have described for the first time the presence of a pneumococcal biofilm-like structure in the lungs of infected mice. We have determined that PsrP mediates a more intimate bacterium to bacterium interaction that contributes to the presence of large bacteria aggregates *in vivo* and increased biofilm biomass and aggregates *in vitro*. This property appears to be shared among other SRRPs including those of medically relevant bacteria such as *S. aureus* and *S. gordonii*, suggesting that it is a conserved function for this class of proteins. How these interactions contribute to pathogenesis remains to be fully determined, however, studies with other bacteria indicate that biofilms serve to inhibit phagocytosis, protect against defensin-mediated killing, and serve as a focal point of infection during early stages of disease. Future experiments will be required to determine the extent to which this may apply for SRRP-mediated aggregates *in vivo*.

## Methods

### Bacterial strains and media

Wild type strains used in this study included *S. pneumoniae* strain TIGR4 and the previously described clinical isolates IPD-5, TNE-6012, and TBE-6050 [8], [12], [14]. T4R is an unencapsulated derivative of TIGR4 [48]. *S. aureus* ISP479C and *S. gordonii* M99 and their corresponding isogenic mutants ISP479C *ΔsraP*, and M99 *ΔgspB* have also been previously described [17], [27]. All of the *S. pneumoniae* mutants used in this study including T4 *ΔpsrP*, T4 *ΩpsrP-secY2A2*, T4 *ΩpsrP*, and T4R *ΩpsrP* have been shown not to have polar effects on upstream and downstream gene transcription [12], [13]. *S. pneumoniae* and *S. gordonii* were grown in Todd-Hewitt broth (THB) or on blood agar plates at 37°C in 5% CO_2_. *S. aureus* were grown in Tryptic-Soy Broth (TSB) or on blood agar plates at 37°C. Stocks for the PsrP mutants were grown in media supplemented with 1 µg/mL of erythromycin, those complemented with the expression vector pNE1 were grown on media supplemented with 250 µg/mL of spectinomycin. SraP and GspB mutant stocks were grown in media supplemented with either 15 µg/mL of erythromycin or 5 µg/mL chloramphenicol respectively. *E. coli* strain DH5α (Invitrogen, Carlsbad CA) expressing recombinant PsrP constructs were grown with 50 µg/mL of kanamycin. Recombinant proteins were purified as previously described [13], [26]. To avoid stress effects on the bacteria, no antibiotics were added to the media during any of the experiments.

### Infection of mice and collection of tissues

Female BALB/cJ mice, 5–6 weeks old, were obtained from The Jackson Laboratory (Bar Harbor, ME). Mice were anesthetized with 2.5% vaporized isoflurane prior to challenge. Exponential phase cultures of *S. pneumoniae* were centrifuged, washed, and suspended in sterile phosphate buffered saline (PBS). For each experimental cohort at least 6 mice were instilled with either 10^7^ cfu of TIGR4 or T4 *ΔpsrP* in 20 µL of PBS into the left nostril. After two days mice were sacrificed for tissue collection. For imaging experiments the intact lungs were collected and processed as described below. For enumeration of bacterial aggregates, nasal lavage fluid was collected from anesthetized mice by instillation and retraction of 20 µl PBS. The same mice were subsequently asphyxiated with compressed CO_2_, and BAL fluid collected by flushing the lungs twice with 0.5 ml of PBS using a sterile catheter. All animal experimentation was conducted following the National Institutes for Health guidelines for housing and care of laboratory animals. Animal experiments were reviewed and approved by the Institutional Animal Care and Use Committee at The University of Texas Health Science Center at San Antonio.

### Scanning electron microscopy (SEM)

Lungs were cut in a sagital orientation, fixed for 2 hours with 2.5% glutaraldehyde in PBS, and then rinsed twice for 3 min in 0.1 M phosphate buffer (pH 7.4). Lungs were submerged in 1% osmium diluted in Zetterquist's Buffer for 30 minutes then washed with the same buffer for 2 minutes [49]. This was followed by step-wise dehydration with ethanol (i.e. 70%, 95%, and 100%); the first two steps for 15 minutes, the last for 30 minutes. Samples were treated with hexamethyldisilizane for 5 minutes prior to drying in a desiccator overnight. The next day samples were sputter coated with gold palladium and viewed with a JEOL-6610 scanning electron microscope.

### Visualization of bacterial aggregates *ex vivo*

From each mouse BAL and 1∶10 PBS diluted nasopharyngeal lavage elutes were smeared onto glass slides, heat fixed, and Gram-stained. Since the nasopharyngeal samples were mucoid, dilution of the samples was warranted. Bacteria were visualized using a CKX41 Olympus microscope at 200× magnification. For each biological sample 100 CFU were randomly selected, taking note of the approximate number of diplococci composing each CFU, either 1, 2–10, or >10. Images of the bacteria were acquired at 400× magnification to better show the multiple bacteria composing the aggregates.

### Fluorescent microscopy of tissue sections

Lung tissues were excised and frozen in Tissue Tek O.C.T solution (Miles Scientific). 5 µm thick lung sections were cut at the University of Texas at San Antonio Histopathology Core and stored at −80° C. Bacteria in the lung sections were detected by immunofluorescence using antibody against the capsular polysaccharide. Sections were thawed, fixed with ice-cold acetone for 20 minutes, and then rehydrated with 70% ethyl alcohol and then PBS. Samples were permeabilized with 0.1% Triton-X-100 for 5 minutes then blocked with 10% fetal bovine serum (FBS) in F12 media for 1 hour. Sections were incubated with 1∶1,000 rabbit anti-serotype 4 pneumococcus antiserum (Statens Serum Institut, Denmark) overnight at 4°C. After washing for three times with 0.5% Tween-PBS, sections were covered with FBS-F12 containing goat anti-rabbit FITC conjugated antibody (Invitrogen) at 1∶2,000 and DAPI (5 µg/ml; for DNA) and the sections incubated for 1 hour at room temperature. Tissue sections were washed and mounted with FluorSave (Merck Biosciences). Images were acquired at 1,000× using a Nikon AX-70 fluorescent Microscope and images processed with SimplePCI software.

### Visualization of early and mature biofilms

Early biofilm formation was examined by measuring the ability of cells to adhere and accumulate biomass on the bottom of a 96-well (flat-bottom) polystyrene plates (Costar, Corning Incorporated, Lowell MA) [24]. Microtiter wells with 200 µl THB were inoculated with 10^6^ CFU of *S. pneumoniae* taken from cultures at mid-logarithmic phase growth (OD_620_ = 0.5). Plates were incubated at 37° C in 5% CO_2_. *S. aureus* and *S. gordonii* biofilm formation on microtiter plates was done in a similar manner, with the exception that TSB was used for *S. aureus* [50], [51]. Bacteria were grown for 2, 4, 6, 8, 18, and 24 h, after which the biofilms were washed gently with PBS and stained with 100 µL of 0.1% CV. Biofilm biomass was subsequently quantified by image capture using an inverted microscope at 15× and 100× magnification and measuring the corresponding optical density (*A*_540_) of the supernatant following washing of the bacteria and solubilization of CV in 200 µL of 95% ethanol.

Mature *S. pneumoniae* biofilms were grown under once through conditions in a glass slide chamber using a continuous-flow through reactor [25]. The flow cell was constructed of anodized aluminum containing a chamber (4.0 mm by 1.3 cm by 5.0 cm) having two glass surfaces, one being a microscope slide and the other being a glass coverslip serving as the substratum. *S. pneumoniae* cells grown to mid-logarithmic phase served as the inoculum and were injected into a septum 4 cm upstream from the flow cell. Bacteria were allowed to attach to the glass substratum for 2 hours prior to initiating flow. The flow rate of the system was adjusted to 0.014 ml/min. Flow through the chamber was laminar, with a Reynolds number of <0.5, having a fluid residence time of 180 min. Biofilms were grown at 37°C in 5% CO_2_ for 3 days under once through conditions. Biofilms were then visualized by confocal laser microscopy as described below.

Biofilms were also grown on the interior surface of a 1-meter long, size 16 Masterflex silicone tubing (0.89mm Internal Diameter, Cole Parmer Inc.) using once-through continuous flow conditions. The line was inoculated with 5 mL of a mid-logarithmic culture and the bacteria were allowed to attach for 2 hours. The flow rate of the system was adjusted to 0.035 ml/min and bacteria were grown for 3 days at 37°C in 5% CO_2_. Bacterial cells were harvested from the interior surface by pinching the tube along its entire length, resulting in removal of the cell material from the lumen of the tubing. Following extraction, exudates were gently suspended in 1 ml of PBS and the optical density (OD_620_) was measured. For light microscopy pictures, 50 µl of line exudate in saline was stained by the addition of 50 µL of 1% CV. A volume of 5 µl of stained line exudates was applied to glass slides, coverslipped, and images taken at 200× magnification using a light microscope. Viable cell counts were determined by plating serial dilutions of exudates following the disruption of each sample by vortexing. Biofilm biomass was determined by measuring the total protein concentration of the exudates by BCA following the complete lysis of *S. pneumoniae* with saline containing 0.1% deoxycholate and 0.1% sodium-dodecyl sulfate, which activates the murein hydrolase autolysin, or use of French press for *S. gordonii* and *S. aureus* cultures. For studies testing whether antibodies or recombinant protein inhibited bacteria aggregation media was supplemented with BR antiserum at 1∶1,000 or spiked with recombinant protein at a final concentration of 1.0 µM.

### Confocal scanning laser microscopy and image acquisition

Confocal scanning laser microscopy was performed with an LSM 510 Meta inverted microscope (Zeiss, Heidelberg, Germany). Images were obtained with an LD-Apochrome 40×/0.6 lens and the LSM 510 Meta image acquisition software (Zeiss). To visualize the biofilm architecture of 3-day-old biofilms, biofilms were stained using the Live/Dead BacLight stain from Invitrogen (Carlsbad, CA). Quantitative analysis of epifluorescence microscopic images obtained from flow cell-grown biofilms at the 6-day time point was performed with COMSTAT image analysis software [52].

### Far Western analysis of BR interactions

Recombinant full-length BR and truncated versions (BR.A, BR.B, BR.C) were expressed and purified from *E. coli* as previously described [13]. Glycosylated PsrP_SRR2(33)-HIS_ was purified in the same manner from TIGR4 (Figure S3), with the exception that cultures were induced with 1% fucose and lysed with 1% SDS in PBS. Far Western analysis was carried out as described by Takamatsu *et al.* with minor modifications [53]. Nitrocellulose membranes were spotted with either 1 µg of whole cell lysate of *S. pneumoniae*, *S. gordonii*, *S. aureus* or *E. coli* expressing various PsrP constructs or with purified recombinant proteins in PBS. Membranes were incubated overnight in PBS with 4% bovine serum albumin and 0.1% Tween 20 (T-PBS) at room temperature. The next day, membranes were washed with T-PBS three times for 5 minutes, and incubated overnight at 4°C on an orbital platform rocker with T-PBS containing 1% bovine serum albumin (TB-PBS) with 1 µg/mL of Gst-BR, PsrP_SRR2(33)-HIS_, or the designated NR constructs from *S. gordonii* and *S. aureus*. Membranes were washed and incubated with monoclonal mouse anti-Gst antibody (1∶5,000 dilution) (Proto-Tech) overnight at 4°C in TB-PBS. Antibody binding was detected by incubating the membranes for 1 h with HRP-conjugated anti-mouse IgG (1∶10,000 dilution) (Sigma), followed by development with the Super Signal chemiluminescent detection system (Thermo Scientific). As a control for inadvertent interactions with the Gst tag, Far Western blots were also performed using an unrelated Gst-tagged *Chlamydia trachomatis* protein (TC0109; Figure S4). No interactions were observed.

### Co-immunoprecipitation of Gst-BR with rBR

Co-immunoprecipitation of Gst-BR with the truncated versions of rPsrP was carried out as previously described by Shivshankar *et al.* [13]. Protein G Sepharose beads (Amersham) were incubated overnight at 4°C with mouse monoclonal penta-His antibody (1∶50; Qiagen) in 500 ml of F12 media supplemented with 10% fetal bovine serum. Beads were incubated with 400 µl of whole bacterial lysates from *E. coli* expressing penta-His tagged recombinant versions of PsrP spiked with 200 µg of recombinant Gst-BR full length and incubated overnight at 4°C with gentle agitation. Beads were washed with RIPA buffer, then boiled in sample buffer for 10 min [54]. Samples were separated on 12% SDS-PAGE gels and electrophoretically transferred to nitrocellulose membranes. Membranes were blocked with T-PBS containing 4% bovine serum for 30 min at room temperature. Membranes were then incubated overnight at 4°C with mouse anti-Gst (1∶7500; Proto-tech) in blocking buffer. Following incubation, membranes were washed with T-PBS three times for 5 minutes. HRP-conjugated goat anti-rabbit Immunoglobulin G (1∶10 000; Sigma) was used as the secondary antibody, followed by development with the Super Signal chemiluminescent detection system (Thermo Scientific).

### Visualization of recombinant BR bound to TIGR4

For labeling of bacteria, TIGR4 and T4 Δ*psrP* were pelleted and suspended in 1 ml of carbonate buffer (pH 9.0) containing FITC (1 mg/ml) and incubated in the dark at room temperature with constant end-to-end tumbling. FITC-labeled bacteria were washed with PBS (pH 7.4) and centrifuged, until the supernatant became clear. rBR fragments were labeled using a FluorLink-Ab Cy3 labeling kit (Amersham) using the instructions provided by the manufacturer. Labeled bacteria were suspended in serum-free F12 media containing the labeled constructs for 1 hour and gently mixed. Subsequently, pneumococci were washed and suspended in F12 medium. Labeled bacteria and bound recombinant protein were visualized using an AX-70 fluorescent microscope and the images were captured at 0.1112–0.8886 ms exposure time for Cy2 and Cy3 filters. The magnification used for capture of digital images was 1000×. Captured images were processed using Simple PCI software.

### Adhesion assays

A549 cells (human alveolar type II pneumocytes; ATCC CRL-185), were grown to 90% confluence on 24-well plates (∼10^6^ cells/well). Prior to use, cells were washed with cell F12 media to remove serum. For competitive inhibition binding assays, A549 cells were incubated with 1µM of either rBR, rBR.C, a synthesized peptide corresponding to AA 122–167, or BSA for 1 HR. Following incubation, cells were exposed to media that contained 10^7^ cfu/mL of bacteria and incubated for 1 h at 37°C in 5% CO_2_. Nonadhering bacteria were removed by washing the cells 3 times with T-PBS and the number of adhering bacteria was determined by lysis of the monolayer with 0.1% Triton X-100 and plating wells per experiment.

### Opsonophagocytosis assay

Bacterial cultures were centrifuged and suspended in 0.1M sodium carbonate buffer (pH 8.0) at an OD_620_ of 0.2. Care was taken to cause minimal disruption of the biofilm aggregates. The diluted cultures were labeled with fluorescent isothiocyanate (1mg/ml) for 30 min at room temperature in the dark. Following labeling, cultures were gently washed three times with sterile PBS to remove free FITC and suspended in PBS. FITC-labeled bacteria were opsonized with 3% control rabbit serum for 30 minutes at 37°C with mild periodical tapping. Mouse J774.1, macrophage cultures maintained in 10% FBS containing DMEM were used for phagocytosis of the opsonized pneumococci. Macrophages were harvested, washed and diluted with opsonophagocytosis buffer (PBS containing 0.2% BSA). FITC-labeled bacteria in 100 µl were added to 10^6^ macrophage cells in 400 µl and incubated for 1 hour at 37°C with periodic shaking. Afterwards, the macrophages were pelleted and washed twice in the assay buffer. Cells were suspended in 400µl of 2% paraformaldehyde until flow cytometric analysis. A2-Laser BD FACSCaliber Analyzer (Becton Dickinson, NJ; Institutional Flow Cytometry Core Facility at the Health Science Center) was employed to analyze percent phagocytic uptake of the labeled bacteria by the macrophages. A minimum of 20,000 events were counted for each sample at 480 nm excitation and 530nm detection wavelengths. Background fluorescence was nullified by subjecting negative control macrophages in assay buffer without any fluorescent bacteria to FACS analysis. Data were processed using CellQuest software.

### Statistical analysis

For pair-wise comparisons of groups statistical analyses were performed using a Student's *t*-test. For multivariate analyses a 1-Way ANOVA followed by a post-priori test using Sigma Stat software was used.

## Supporting Information

Figure S1 Immunofluorescent imaging of *S. pneumoniae* in the alveolar space. Frozen lung sections from TIGR4 and T4 Δ*psrP* infected mice were processed for imaging using anti-serotype 4 capsular antiserum (Statens Serum Institute; Denmark). Images were taken at 600× and are representative. Note the detection of a large bacterial aggregate (green) for TIGR4 infected animals, whereas for mice infected with T4 Δ*psrP* smaller clusters are evident.
(0.12 MB PDF)

Figure S2 Deletion of *psrP-secY2A2* alters bacteria interactions in mature biofilms. **A**) Micrographs of mature TIGR4 and T4 *ΩpsrP-secY2A2* biofilms. Bacteria were grown in THB at 37° C in 5% CO_2_ on glass slides within a flow cell under once-through flow conditions for 3 days. For visualization, bacteria were stained with Live/Dead BacLight stain. Biofilms were viewed at 400× magnification using an inverted confocal laser scanning microscope. **B**) Quantitative analysis of biofilms was performed using COMSTAT image analysis software. Flow cell experiments were performed in triplicate. Statistical analyses were performed using a two-tailed Student's *t*-test. For panel C error bars denote standard error.
(0.26 MB PDF)

Figure S3 Illustration of the *psrP* loci in TIGR4 & T4R, T4 Ω*psrP &* T4R Ω*psrP*, and assorted pNE1 plasmids encoding truncated versions of *psrP*.
(0.08 MB PDF)

Figure S4 Far Western analyses using a GST tagged protein (TC0109) from *Chlamydia trachomatis* as a probe for non-specific interactions due to the Gst-tag. Membranes were spotted with either **A**) lysates from *S. pneumoniae* expressing truncated versions of PsrP; **B**) truncated versions of His tagged rBR expressed and purified from *E. coli*; or **C**) whole cell lysates from *S. gordonii*, *S. aureus*, *S. pneumoniae* and their respective isogenic SRRP mutants.
(0.22 MB PDF)

Figure S5 Purification of a glycosylated PsrP construct. **A**) Illustration of the *psrP_SRR2(33)-HIS_* locus in the expression vector pNE1. The plasmid was used to express and purify glycosylated PsrP from *S. pneumoniae*, strain TIGR4 cell lysates. Note the presence of *fcsRK*, a pneumococcal fucose-inducible promoter, also that the cell wall anchor domain has been replaced with a 6× histidine tag. **B**) Western blot of glycosylated PsrP_SRR2(33)-HIS_ in TIGR4 following induction with 1% fucose. Despite having a predicted mass of 66 kDa PsrP_SRR2(33)-HIS_ separates at an apparent molecular mass of 200 kDa. This is due to glycosylation and these findings are consistent with earlier work by Shivshankar *et al.* [13].
(0.08 MB PDF)

Figure S6 Antiserum against TIGR4 BR inhibits biofilm formation of unrelated clinical isolates that carry PsrP. Low passage clinical isolates of *S. pneumoniae* carrying PsrP (TNE-6050, TNE 6012) or without (IPD-5), were grown in silicone coated lines under once-through conditions at 37°C in 5% CO_2_ for 3 days. THB was supplemented with either naïve rabbit serum (control) or antiserum to recombinant TIGR4 BR at a dilution of 1∶1000. Following incubation, biofilms were extruded and analyzed. **A**) Micrographs of CV stained bacteria extruded from the biofilm lines. **B**) Optical density (OD_540_) of bacterial exudates. **C**) Levels of protein in bacteria line exudates as determined by BCA analysis. Note that antiserum against BR did not affect biofilm formation by IPD-5, the PsrP deficient clinical isolate. Images are representative of at least 3 independent experiments. Statistical analyses were performed using a two-tailed Student's *t*-test. Error bars denote standard error. Asterisks denote statistical significance versus whole sera.
(0.26 MB PDF)
