# Supplementary material for: The Pneumococcal Serine-Rich Repeat Protein Is an Intra-Species Bacterial Adhesin That Promotes Bacterial Aggregation In Vivo and in Biofilms
Source: PLoS Pathog. 2010 Aug 12;6(8):e1001044. doi: 10.1371/journal.ppat.1001044 (PMC2920850; doi:10.1371/journal.ppat.1001044)
Supplement: Figure S1 — Immunofluorescent imaging of S. pneumoniae in the alveolar space. Frozen lung sections from TIGR4 and T4 ΔpsrP infected mice were processed for imaging using anti-serotype 4 capsular antiserum (Statens Serum Institute; Denmark). Images were taken at 600× and are representative. Note the detection of a large bacterial aggregate (green) for TIGR4 infected animals, whereas for mice infected with T4 ΔpsrP smaller clusters are evident. (0.12 MB PDF) [file ppat.1001044.s001.pdf]

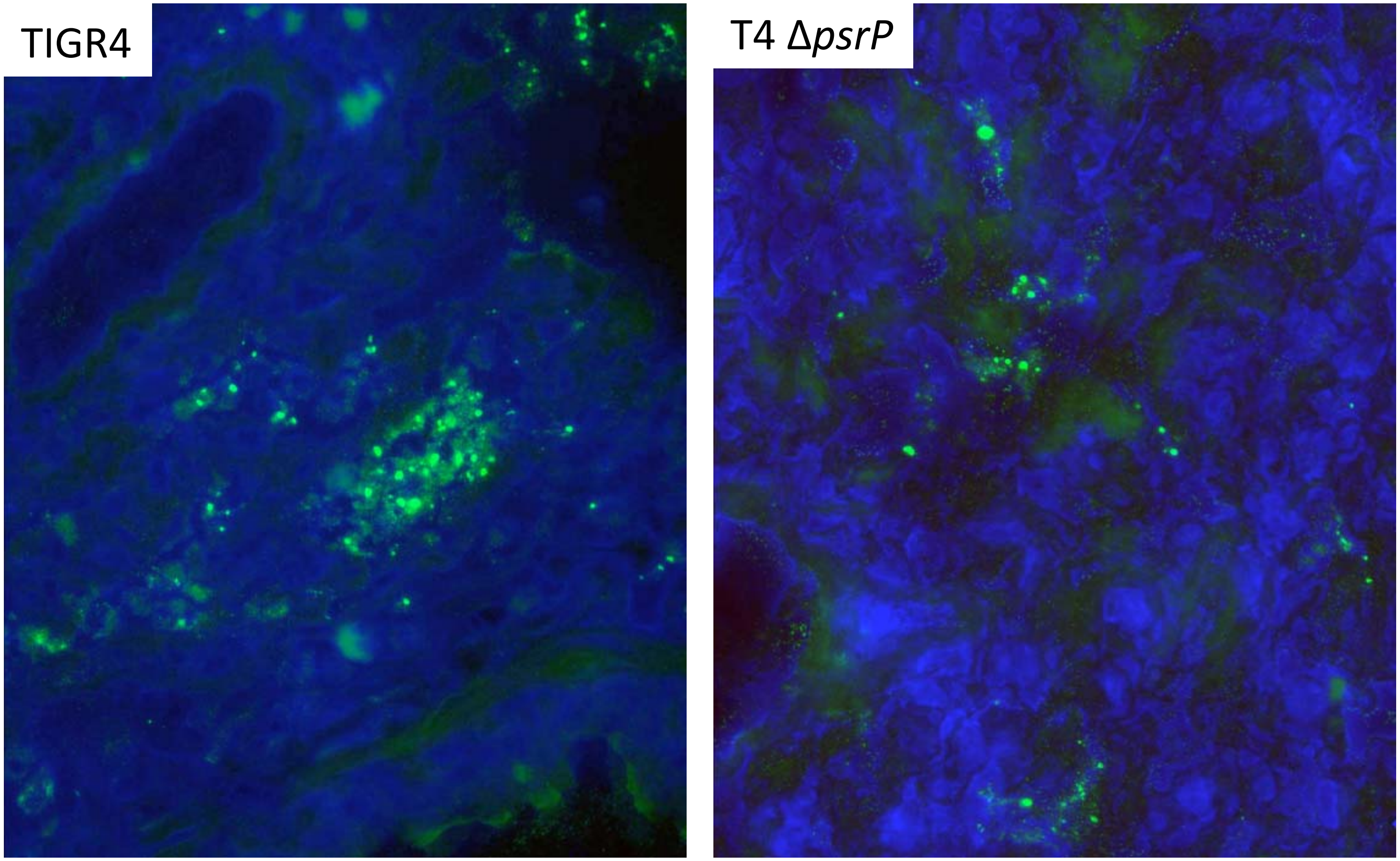

Figure S1. Immunofluorescent imaging of *S. pneumoniae* in the alveolar space. Frozen lung sections from TIGR4 and T4 Δ*psrP* infected mice were processed for imaging using anti-serotype 4 capsular antiserum (Statens Serum Institute; Denmark). Images were taken at 600X and are representative. Note the detection of a large bacterial aggregate (green) for TIGR4 infected animals, whereas for mice infected with T4 Δ*psrP* smaller clusters are evident.
