# Supplementary material for: The Pneumococcal Serine-Rich Repeat Protein Is an Intra-Species Bacterial Adhesin That Promotes Bacterial Aggregation In Vivo and in Biofilms
Source: PLoS Pathog. 2010 Aug 12;6(8):e1001044. doi: 10.1371/journal.ppat.1001044 (PMC2920850; doi:10.1371/journal.ppat.1001044)
Supplement: Figure S2 — Deletion of psrP-secY2A2 alters bacteria interactions in mature biofilms. A) Micrographs of mature TIGR4 and T4 ΩpsrP-secY2A2 biofilms. Bacteria were grown in THB at 37° C in 5% CO2 on glass slides within a flow cell under once-through flow conditions for 3 days. For visualization, bacteria were stained with Live/Dead BacLight stain. Biofilms were viewed at 400× magnification using an inverted confocal laser scanning microscope. B) Quantitative analysis of biofilms was performed using COMSTAT image analysis software. Flow cell experiments were performed in triplicate. Statistical analyses were performed using a two-tailed Student's t-test. For panel C error bars denote standard error. (0.26 MB PDF) [file ppat.1001044.s002.pdf]

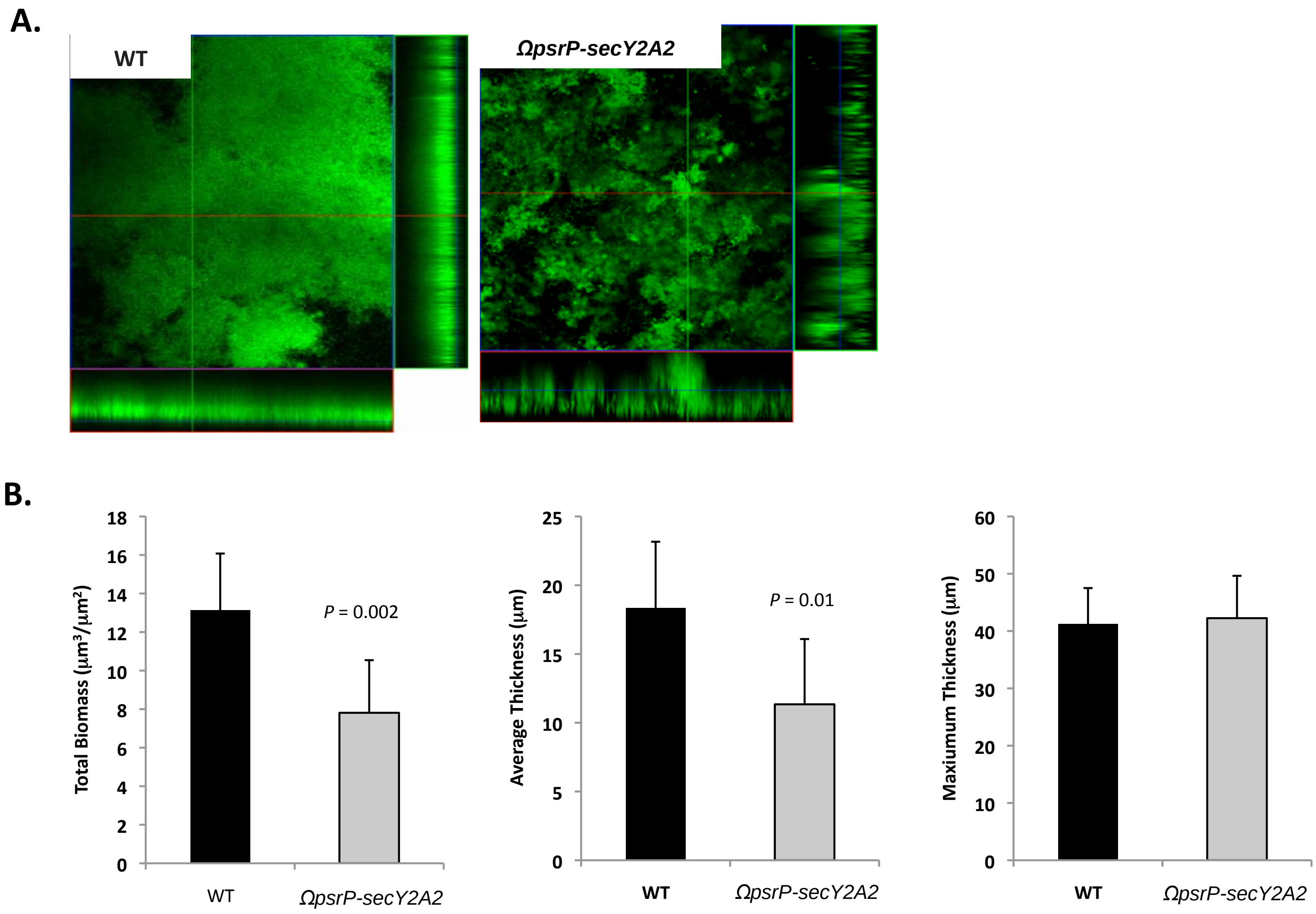

Figure S2. Deletion of *psrP-secY2A2* alters bacteria interactions in mature biofilms. A) Micrographs of mature TIGR4 and T4 *ΩpsrP-secY2A2* biofilms. Bacteria were grown in THB at 37° C in 5% CO_2 on glass slides within a flow cell under once-through flow conditions for 3 days. For visualization, bacteria were stained with Live/Dead BacLight stain. Biofilms were viewed at 400X magnification using an inverted confocal laser scanning microscope. **B)** Quantitative analysis of biofilms was performed using COMSTAT image analysis software. Flow cell experiments were performed in triplicate. Statistical analyses were performed using a two-tailed Student's *t*-test. For panel C error bars denote standard error.
