# Supplementary material for: The Pneumococcal Serine-Rich Repeat Protein Is an Intra-Species Bacterial Adhesin That Promotes Bacterial Aggregation In Vivo and in Biofilms
Source: PLoS Pathog. 2010 Aug 12;6(8):e1001044. doi: 10.1371/journal.ppat.1001044 (PMC2920850; doi:10.1371/journal.ppat.1001044)
Supplement: Figure S3 — Illustration of the psrP loci in TIGR4 & T4R, T4 ΩpsrP & T4R ΩpsrP, and assorted pNE1 plasmids encoding truncated versions of psrP. (0.08 MB PDF) [file ppat.1001044.s003.pdf]

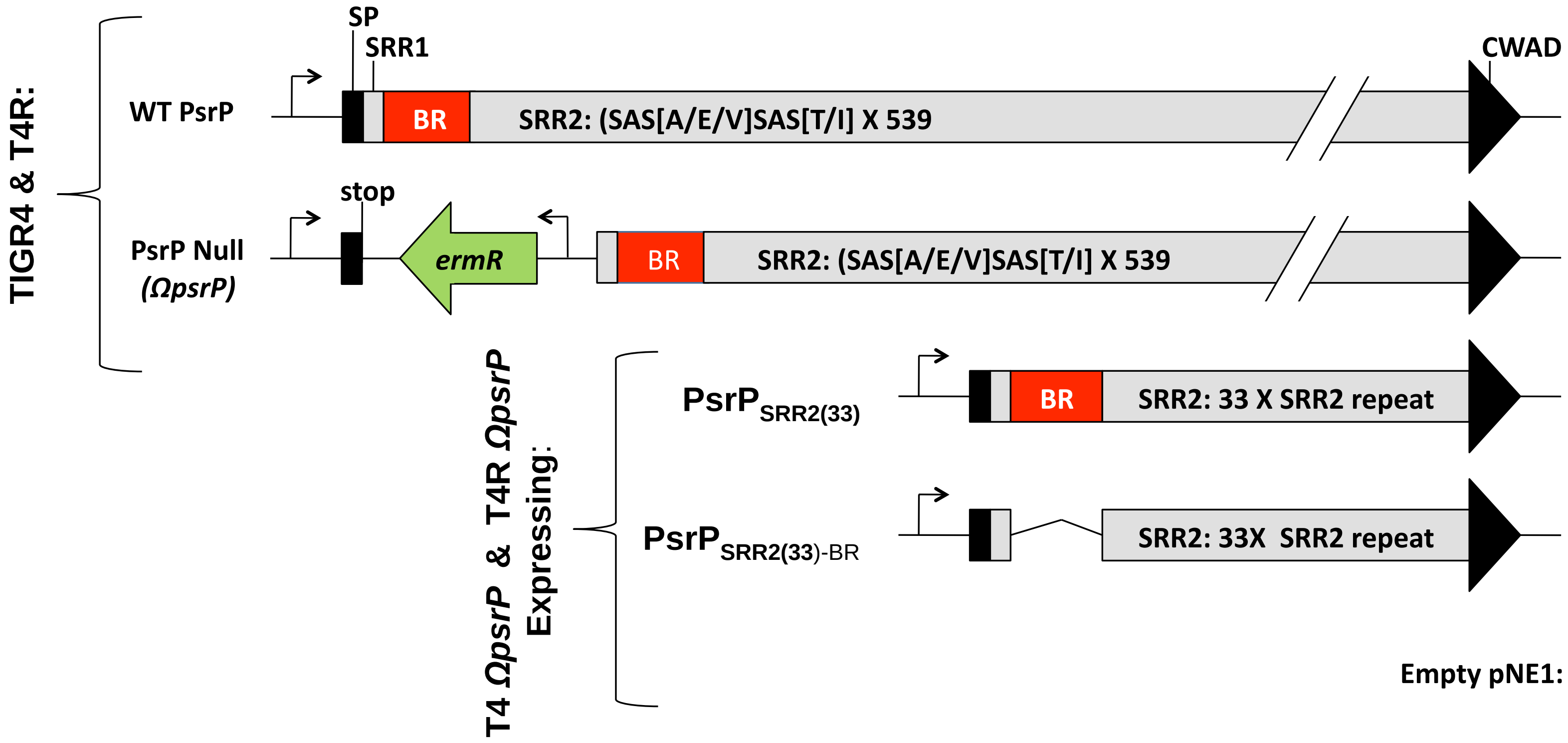

Figure S3. Illustration of the *psrP* loci in TIGR4 & T4R, T4 Ω*psrP* & T4R Ω*psrP*, and assorted pNE1 plasmids encoding truncated versions of *psrP.*
