# Supplementary material for: The Pneumococcal Serine-Rich Repeat Protein Is an Intra-Species Bacterial Adhesin That Promotes Bacterial Aggregation In Vivo and in Biofilms
Source: PLoS Pathog. 2010 Aug 12;6(8):e1001044. doi: 10.1371/journal.ppat.1001044 (PMC2920850; doi:10.1371/journal.ppat.1001044)
Supplement: Figure S4 — Far Western analyses using a GST tagged protein (TC0109) from Chlamydia trachomatis as a probe for non-specific interactions due to the Gst-tag. Membranes were spotted with either A) lysates from S. pneumoniae expressing truncated versions of PsrP; B) truncated versions of His tagged rBR expressed and purified from E. coli; or C) whole cell lysates from S. gordonii, S. aureus, S. pneumoniae and their respective isogenic SRRP mutants. (0.22 MB PDF) [file ppat.1001044.s004.pdf]

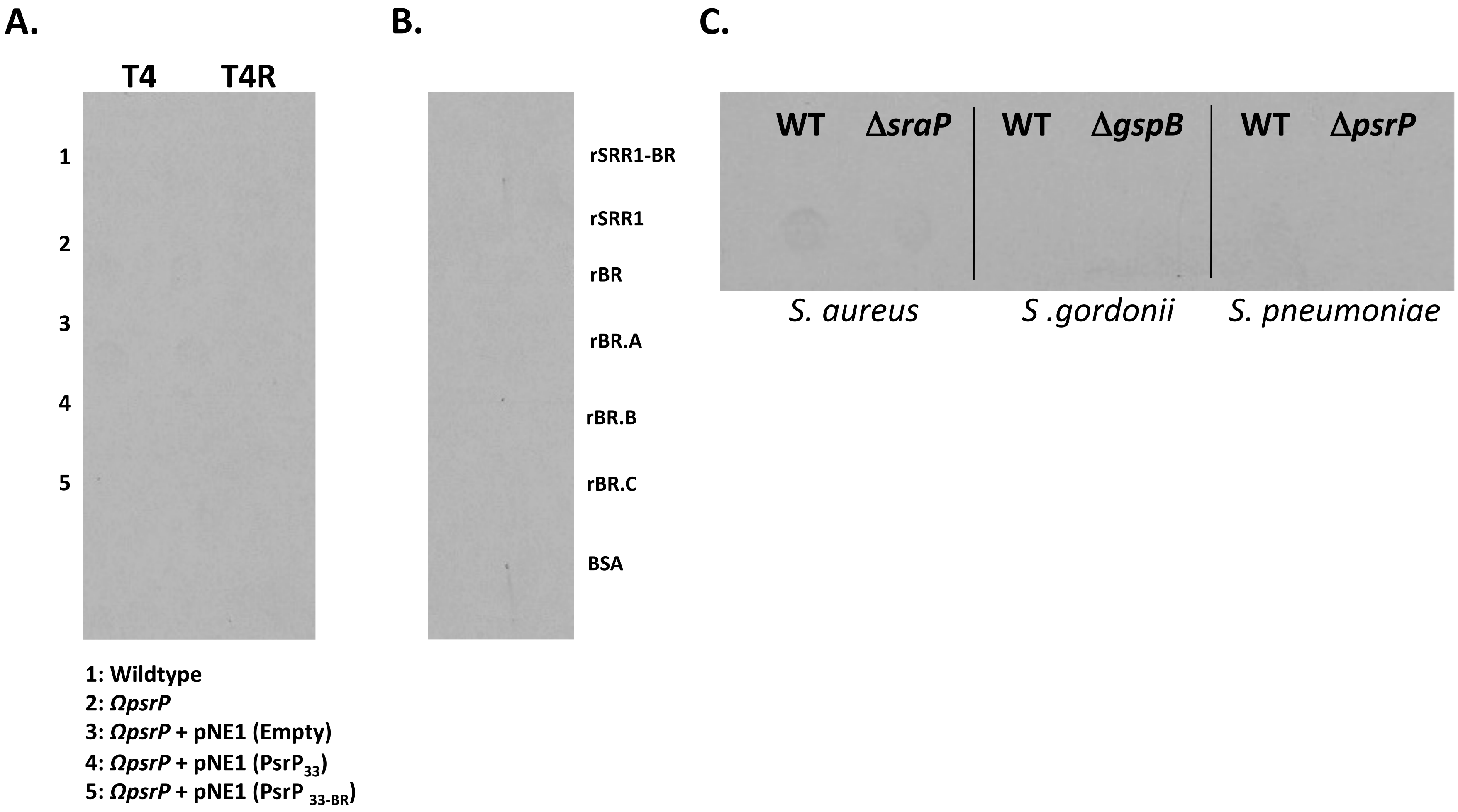

Figure S4. Far Western analyses using a GST tagged protein (TC0109) from *Chlamydia trachomatis* as a probe for non-specific interactions due to the Gst-tag. Membranes were spotted with either **A)** lysates from *S. pneumoniae* expressing truncated versions of PsrP; **B)** truncated versions of His tagged rBR expressed and purified from *E. coli*; or **C)** whole cell lysates from *S. gordonii*, *S. aureus, S. pneumoniae* and their respective isogenic SRRP mutants.
