# Supplementary material for: The Pneumococcal Serine-Rich Repeat Protein Is an Intra-Species Bacterial Adhesin That Promotes Bacterial Aggregation In Vivo and in Biofilms
Source: PLoS Pathog. 2010 Aug 12;6(8):e1001044. doi: 10.1371/journal.ppat.1001044 (PMC2920850; doi:10.1371/journal.ppat.1001044)
Supplement: Figure S5 — Purification of a glycosylated PsrP construct. A) Illustration of the psrPSRR2(33)-HIS locus in the expression vector pNE1. The plasmid was used to express and purify glycosylated PsrP from S. pneumoniae, strain TIGR4 cell lysates. Note the presence of fcsRK, a pneumococcal fucose-inducible promoter, also that the cell wall anchor domain has been replaced with a 6× histidine tag. B) Western blot of glycosylated PsrPSRR2(33)-HIS in TIGR4 following induction with 1% fucose. Despite having a predicted mass of 66 kDa PsrPSRR2(33)-HIS separates at an apparent molecular mass of 200 kDa. This is due to glycosylation and these findings are consistent with earlier work by Shivshankar et al. [13]. (0.08 MB PDF) [file ppat.1001044.s005.pdf]

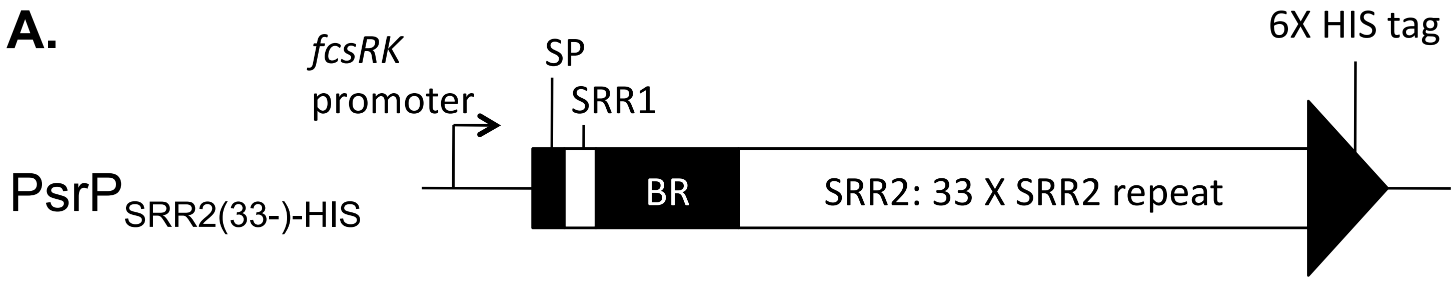

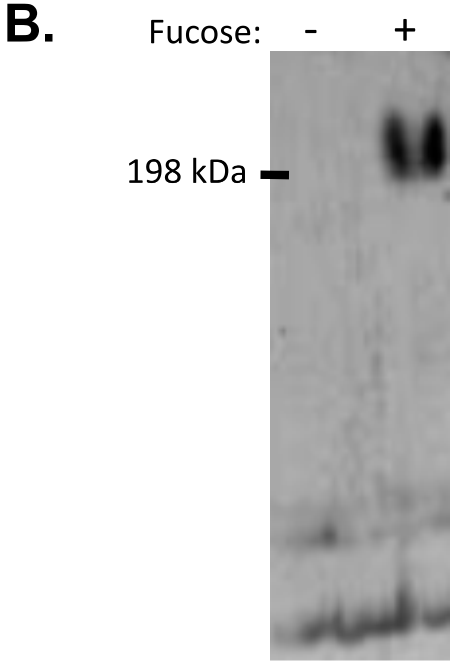

Figure S5. Purification of a glycosylated PsrP construct. A) Illustration of the $psrP_{SRR2(33)\text{-}HIS}$ locus in the expression vector pNE1. The plasmid was used to express and purify glycosylated PsrP from *S. pneumoniae,* strain TIGR4 cell lysates. Note the presence of *fcsRK*, a pneumococcal fucose-inducible promoter. Also that the cell wall anchor domain has been replaced with a 6X histidine tag. **B)** Western blot of glycosylated $PsrP_{SRR2(33)\text{-}HIS}$ in TIGR4 following induction with 1% fucose. Despite having a predicted mass of 66 kDa, due to to glycosylation, $PsrP_{SRR2(33)\text{-}HIS}$ separates at an apparent molecular mass of 200 kDa. These findings are consistent with earlier work (Shivshankar *et al.* 2009).
