# Supplementary material for: The Pneumococcal Serine-Rich Repeat Protein Is an Intra-Species Bacterial Adhesin That Promotes Bacterial Aggregation In Vivo and in Biofilms
Source: PLoS Pathog. 2010 Aug 12;6(8):e1001044. doi: 10.1371/journal.ppat.1001044 (PMC2920850; doi:10.1371/journal.ppat.1001044)
Supplement: Figure S6 — Antiserum against TIGR4 BR inhibits biofilm formation of unrelated clinical isolates that carry PsrP. Low passage clinical isolates of S. pneumoniae carrying PsrP (TNE-6050, TNE 6012) or without (IPD-5), were grown in silicone coated lines under once-through conditions at 37°C in 5% CO2 for 3 days. THB was supplemented with either naïve rabbit serum (control) or antiserum to recombinant TIGR4 BR at a dilution of 1∶1000. Following incubation, biofilms were extruded and analyzed. A) Micrographs of CV stained bacteria extruded from the biofilm lines. B) Optical density (OD540) of bacterial exudates. C) Levels of protein in bacteria line exudates as determined by BCA analysis. Note that antiserum against BR did not affect biofilm formation by IPD-5, the PsrP deficient clinical isolate. Images are representative of at least 3 independent experiments. Statistical analyses were performed using a two-tailed Student's t-test. Error bars denote standard error. Asterisks denote statistical significance versus whole sera. (0.26 MB PDF) [file ppat.1001044.s006.pdf]

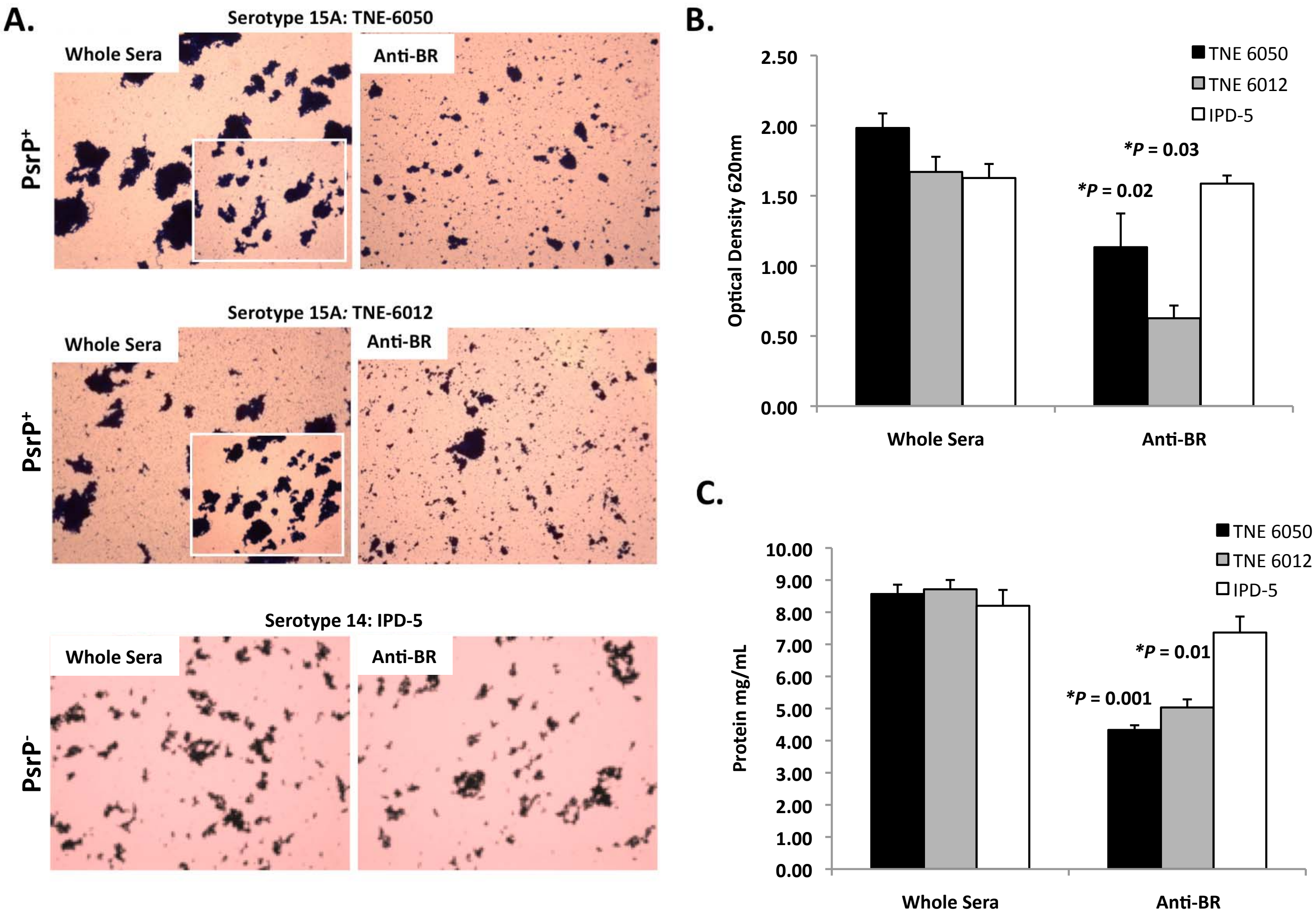

Figure S6. Antiserum against TIGR4 BR inhibits biofilm formation of unrelated clinical isolates that carry PsrP. Low passage clinical isolates of *S. pneumoniae* carrying PsrP (TNE-6050, TNE 6012) or without (IPD-5), were grown in silicone coated lines under once-through conditions at 37°C in 5% CO_2 for 3 days. THB was supplemented with either naïve rabbit serum (control) or antiserum to recombinant TIGR4 BR at a dilution of 1:1000. Following incubation, biofilms were extruded and analyzed. **A)** Micrographs of CV stained bacteria extruded from the biofilm lines. **B)** Optical density (OD_{540}) of bacterial exudates. **C)** Levels of protein in bacteria line exudates as determined by BCA analysis. Note that antiserum against BR did not affect biofilm formation by IPD-5, the PsrP deficient clinical isolate. Images are representative of at least 3 independent experiments. Statistical analyses were performed using a two-tailed Student's *t*-test. Error bars denote standard error. Asterisks denote statistical significance versus whole sera.
